# Tomatoes

**Published:** 1871-10

**Authors:** 


### TOMATOES

Are destroyed by the first frosts; if the vines are taken up, with half a bushel of earth around them, and thus removed to the cellar, they will continue to ripen until the beginning of winter; we have eaten watermelons at Christmas, thus kept to ripen; the vines should be spread out, so as to get the air, and the cellar should be warm enough to exclude frost.

